# Correction: Replicating the Disease framing problem during the 2020 COVID-19 pandemic: A study of stress, worry, trust, and choice under risk

**DOI:** 10.1371/journal.pone.0315440

**Published:** 2024-12-05

**Authors:** The PLOS One Staff

The first four authors should have not been marked equally contributed on this work. Nikolay R. Rachev and Hyemin Han are co-first authors of this work. David Lacko and Rebekah Gelpí are co-second authors of this work.

In the heading The framing effect and the disease problem subsection of Introduction, there is an error in the capitalization of disease. The correct heading is: The framing effect and the Disease problem.

In the heading Risky choice and the framing effect: COVIDiSTRESS vs. the many labs data, there is an error in the capitalization of many labs. The correct heading is: Risky choice and the framing effect: COVIDiSTRESS vs. the Many Labs data.

The publisher apologizes for the errors.

## References

[1] Rachev NR, Han H, Lacko D, Gelpí R, Yamada Y, Lieberoth A (2021) Replicating the Disease framing problem during the 2020 COVID-19 pandemic: A study of stress, worry, trust, and choice under risk. PLoS ONE 16(9): e0257151. 10.1371/journal.pone.0257151 34506543 PMC8432807

