# Supplementary material for: Replicating the Disease framing problem during the 2020 COVID-19 pandemic: A study of stress, worry, trust, and choice under risk
Source: PLoS One. 2021 Sep 10;16(9):e0257151. doi: 10.1371/journal.pone.0257151 (PMC8432807; doi:10.1371/journal.pone.0257151)

Replicating the Disease framing problem during the 2020 COVID-19 pandemic: A study of stress, worry, trust, and choice under risk

S1 File

Supplementary Methods

Hypothesis testing

Hypothesis 1a

We first tested the proportion of "safe" choices on the ADP, (*n* of participants selected Program A + *n* of participants selected Program C) / *N* of participants, separately for both Klein et al.'s (2014) dataset and for our dataset. We used frequentist and Bayesian MLM to compare the risk aversion tendency between Klein et al. (2014) and our study. We first examined the following four models, reported using the R package *lme4* notation:

Model 0 (Null model only with intercept): risk_aversion ~ 1

Model 1 (Model with an added random intercept): risk_aversion ~ 1 + (1 | country)

Model 2 (Model with the independent variable of interest and random intercept): risk_aversion ~ study + (1 | country)

Model 3: (Model with random slopes): risk_aversion ~ study + (1 + study || country)

In this model, all variables to be analyzed, the risk aversion and study assignment, were individual-level variables.

With the *lmer* function from the *R* package *lme4*, we compared which model best fitted both datasets in terms of AIC and BIC. One the best model was identified, we examined whether the main effect of the study was significantly greater than zero. For Bayesian MLM, *brms* package was utilized (Han, Park, & Thoma, 2018; Keysers, Gazzola, & Wagenmakers, 2020). Whether the main effect of the study was significantly greater than zero was examined with the resultant Bayes Factor. For the prior, we used the default Cauchy prior distribution for regression analysis, Cauchy (0, 1) (Rouder & Morey, 2012). Once the Bayes Factor was calculated, we examined whether the resultant Bayes Factor exceeded the threshold for at least positive evidence supporting the alternative hypothesis (BF ≥ 3).

Hypothesis 1b

To examine whether the framing effect in our study was greater than that reported in Klein et al. (2014), we performed multilevel logistic regression. Our dataset was analyzed with frequentist and Bayesian MLM. These four models were compared:

Model 0 (Null model only with intercept): choice ~ 1

Model 1 (Model with an added random intercept): choice ~ 1 + (1 | country)

Model 2 (Model with the independent variable of interest and random intercept): choice ~ frame + (1 | country)

Model 3: (Full model with random slopes): choice ~ frame + (1 + frame || country)

In this model, all variables to be analyzed, the choice and presented frame, were individual-level variables.

With the *lmer* function from the *R* package *lme4*, we compared which model best fitted both datasets in terms of AIC and BIC. Then, we examined the main effect of frame.

For the comparison of the effect size of framing in our study vs. Klein et al. (2014), we employed Bayesian multilevel logistic regression (Han et al., 2018; Keysers, Gazzola, & Wagenmakers, 2020) using *R* package *brms*. Once *brms* completed and the posterior of $d_{current}$, the estimated effect size of frame in our study, was estimated, we examined "$d_{Klein} < d_{current}$." d_{Klein} was the effect size of framing effect reported in Klein et al.'s (2014) study, Cohen's $d = .60$. We examined whether the resultant Bayes Factor exceeded the threshold for at least positive evidence supporting the alternative hypothesis (BF $\geq$ 3).

Hypothesis 2a to 2f

To test whether higher levels of distress (as measured by PSS-10) predicted the proportion of risky choices on ADP, we used multilevel logistic regression using the *glmer* function (with the "family" parameter set to "binomial"). We performed the following model comparisons using the following steps (Sommet & Morselli, 2017):

Model 0 (Null model only with intercept): choice ~ 1

Model 1 (Model with an added random intercept): choice ~ 1 + (1 | country)

Model 2 (Model with the independent variable of interest and random intercept): choice ~ frame * PSS-10 + (1 | country)

Model 3 (Full model with random slopes): choice ~ frame * PSS-10 + (1 + PSS-10 || country) + (1 + frame || country)

In this model, all variables to be analyzed, the choice, presented frame, and PSS-10, were individual-level variables.

We interpreted the results from whichever among Model 2 and Model 3 fitted the data better in terms of AIC, BIC, and Bayes Factors. We interpreted a significant main effect of PSS-10 (PSS-10 > 0) as evidence in favor of H2a. We interpreted significant positive interaction terms as evidence in favor of H2b if the interaction was as predicted. Given that our hypotheses were directional, we used one-tail tests for our frequentist analyses.

In addition to the frequentist tests with *glmer*, we performed Bayesian multilevel logistic regression with *brms* to examine whether evidence supported H2a and H2b. Bayesian MLM was performed only when we found significant outcomes from frequentist MLM that allowed us to reject the null hypothesis. We ran *brms* with the same *glmer* models indicated from the processes described above, searched for the best model fit, and examined resultant Bayes Factors. We used Cauchy priors, Cauchy (d = 0, scale = 1) for estimating coefficients as suggested by Rouder and Morey (2012). For H2a, we investigated whether the Bayes Factor corresponding to the main effect of PSS_10 > 0 was at least ≥ 3 (positive evidence) or ≥ 10 (strong evidence). For H2b, we investigated whether the Bayes Factor corresponding to the interaction effect > 0 was at least ≥ 3 (positive evidence) or ≥ 10 (strong evidence).

To test hypotheses 2c and d and 2e and f, we repeated the same steps by replacing "PSS_10" with "coronavirus concerns" and "trust", respectively. Similar to the aforementioned testing, both coronavirus concerns and trust were individual-level variables.

Hypothesis 3a and 3b

To test whether risk seeking and framing varied by country, we examined the following multilevel models:

Model 0 (Null model only with intercept): choice ~ 1

Model 1 (Model with an added random intercept): choice ~ 1 + (1 | country)

Model 2 (Model with the independent variable of interest and random intercept): choice ~ country + (1 | country)

Model 3 (Model also including the effect of frame and the frame * country interaction): choice ~ frame * country + (1 | country)

In this model, all variables to be analyzed, the choice and presented frame, were individual-level variables.

The same frequentist and Bayesian MLM procedures and criteria provided in the prior section were applied. Frequentist analysis was performed with *glmer* and models specified above. The same models were examined with *brms* for Bayesian analysis. Bayesian MLM was performed only when we found significant outcomes from frequentist MLM that allowed us to reject the null hypothesis. Once the best model was identified, we examined whether the main effect of country (H3a) and interaction effect of frame x country (H3b) were significantly different from zero with frequentist MLM and Bayesian MLM (Bayes Factor ≥ 3).

To examine the significance of the frame × country interaction in Model 3, we tested whether the inclusion of the interaction effect significantly improved the model. In order to do so, we performed MLM with one additional model, Model 2.5:

Model 2.5 (Model 2 + the main effect of frame): choice ~ frame + country + (1 | country)

Then, we compared Models 3 and 2.5. In the case of frequentist analysis, we performed the Type III Wald χ^2 test, which was implemented in *lmerTest*. Given that Models 3 and 2.5 were nested, by performing this likelihood ratio test, it was possible to test whether the inclusion of the interaction effect significantly improved the model. In the case of Bayesian MLM, we compared Bayes Factors of Model 3 vs. Model 2.5.

Exploratory analyses

For each additional exploratory analysis, we tested these models:

Supplementary Analysis on Hypothesis 2c and 2d:

Model 0: Safe choice ~ 1 (only with an intercept)

Model 1: Safe choice ~ 1 + (1 | Country) (with the random intercepts of the country)

Model 2: Safe choice ~ Concerns + (1 | County) (with the main effect of concerns)

Model 3: Safe choice ~ Concerns + (1 + Concerns | County) (with the random slopes of the country)

In this model, the coronavirus concerns estimate was an individual-level variable.

GDP per Capita, Risky Choice, and the Framing Effect:

GDP per capita data was acquired at the country level. The data was converted into a log scale for analysis.

Model 0 (Null model only with intercept): choice ~ 1

Model 1 (Model with an added random intercept): choice ~ 1 + (1 | country)

Model 2 (Model with the independent variable of interest and random intercept): choice ~ frame * log(GDP per capita) + (1 | country)

Model 3 (Full model with random slopes): choice ~ frame * log(GDP per capita) + (1 + log(GDP per capita) || country) + (1 + frame || country)

Other than GDP per capita, the choice and presented frame were individual-level variables

Compliance, Risky Choice, and the Framing Effect:

Model 0 (Null model only with intercept): choice ~ 1

Model 1 (Model with an added random intercept): choice ~ 1 + (1 | country)

Model 2 (Model with the independent variable of interest and random intercept): choice ~ frame * compliance + (1 | country)

Model 3 (Full model with random slopes): choice ~ frame * compliance + (1 + compliance || country) + (1 + frame || country)

In this model, all variables to be analyzed, the choice, presented frame, and compliance, were individual-level variables.

Familiarity with the Asian Disease Problem, Risky Choice, and the Framing Effect:

Model 0 (Null model only with intercept): choice ~ 1

Model 1 (Model with an added random intercept): choice ~ 1 + (1 | country)

Model 2 (Model with the independent variable of interest and random intercept): choice ~ frame * familiarity + (1 | country)

Model 3 (Full model with random slopes): choice ~ frame * familiarity + (1 + familiarity || country) + (1 + frame || country)

In this model, all variables to be analyzed, the choice, presented frame, and familiarity with the problem, were individual-level variables.

To answer the aforementioned three questions, identical to the planned hypothesis testing, we performed both frequentist and Bayesian MLM. The same model selection and result interpretation procedures were applied. In the tested models, we assumed uncorrelated random effects due to the computational complexity and load involved in Bayesian MLM.

Supplementary Tables

Table S1.1

Deviations from the Preregistered Protocol

Category	Preregistered	Conducted	Justification
Participants: Exclusion criteria	Include countries with more than 30 participants	Included countries with more than 100 participants	A desirable threshold for the measurement invariance tests
Criteria for model comparison	(Not specified)	For metric invariance, -.01 change in CFI, +.015 in RMSEA, and +.030 in SRMR.<br>For scalar invariance, -.01 change in CFI,	Criteria for measurement invariance test not described

		+.015 in RMSEA, and +.015 in SRMR	
	Compare which model best fits both datasets in terms of AIC, BIC, and aBIC	aBIC not used	Lack of functionality to calculate aBIC in MLM implemented in R
Data analysis	R or MPlus	R only	For better accessibility and further replicability and reproductivity
Hypothesis testing	(Directions of tests not specified in the analysis plans although the directions were specified in the hypotheses)	One-tailed tests used	Hypotheses are directional but such a point was not well applied in the analysis plans
PSS Factor Structure	Use the two-factor model of PSS	Tested the two-factor model as well as the one-factor model	Measurement alignment could be implemented only with a one-factor model
Supplementary exploratory analysis on Hypotheses 2c and 2d	(Not specified)	The supplementary exploratory analysis was conducted.	The decision was data-driven.

Table S1.2

Supplementary Analysis on Hypothesis 2c and 2d: Model Comparison

Model	AIC	BIC
0	122,147.50	122,156.90
1	121,235.70	121,254.40
2	**121,234.80**	**121,263.00**
3	121,237.80	121,284.70

Note. Numbers in bold represent the best model for the respective hypothesis.

Supplementary Figures

Figure S1.1

Relationship between the predicted probability of the safe choice and the frame moderated by the distress (H2ab)

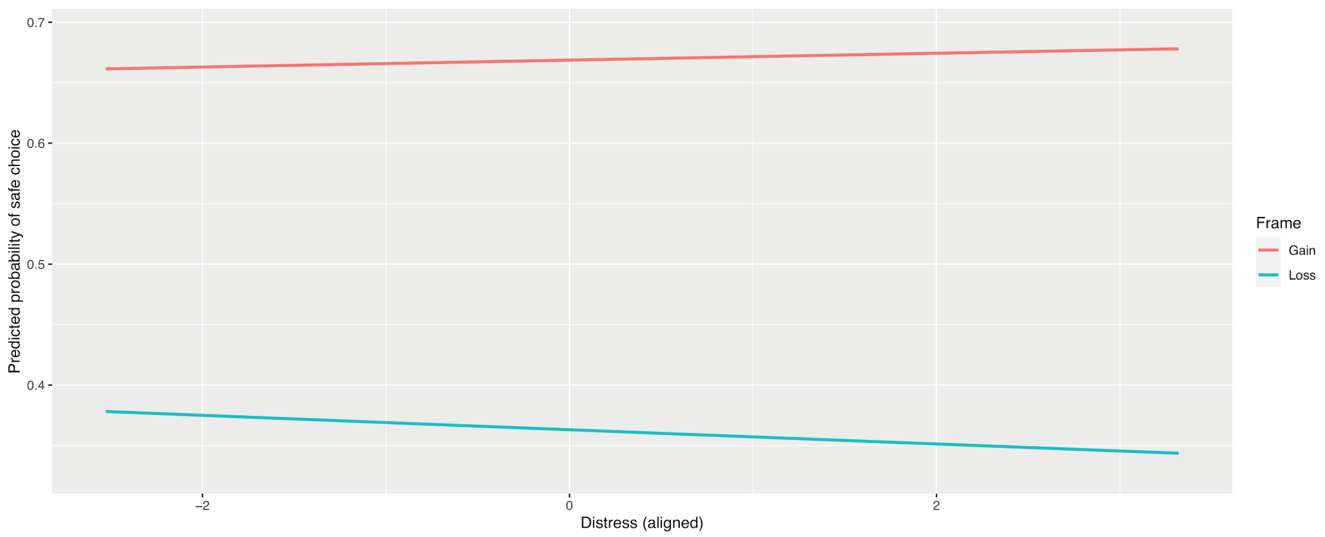

Figure S1.2

Relationship between the predicted probability of the safe choice and the frame moderated by coronavirus concerns (H2cd)

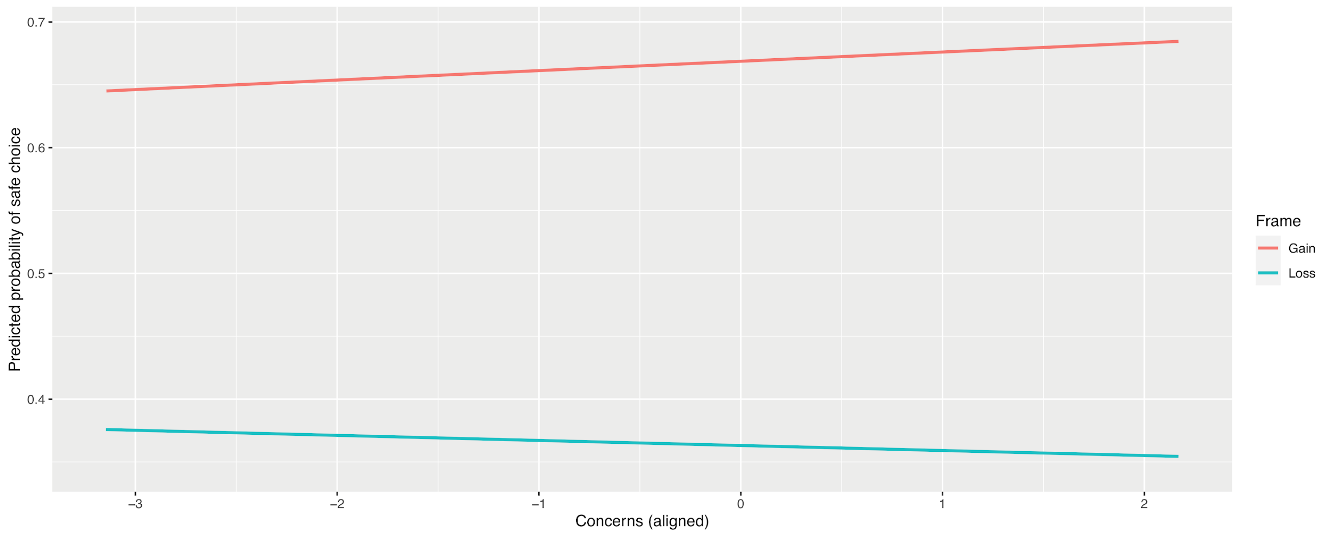

Figure S1.3

Relationship between the predicted probability of the safe choice and the frame moderated by trust in government efforts (H2ef)

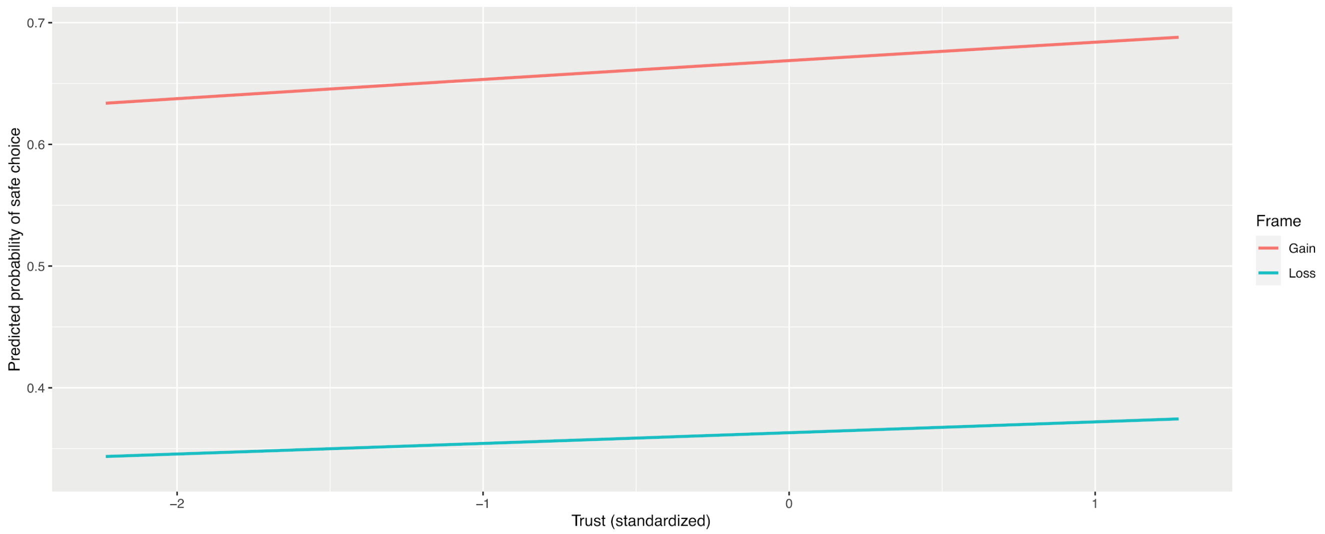

Figure S1.4

Relationship between the predicted probability of the safe choice and the frame moderated by log GDP per capita

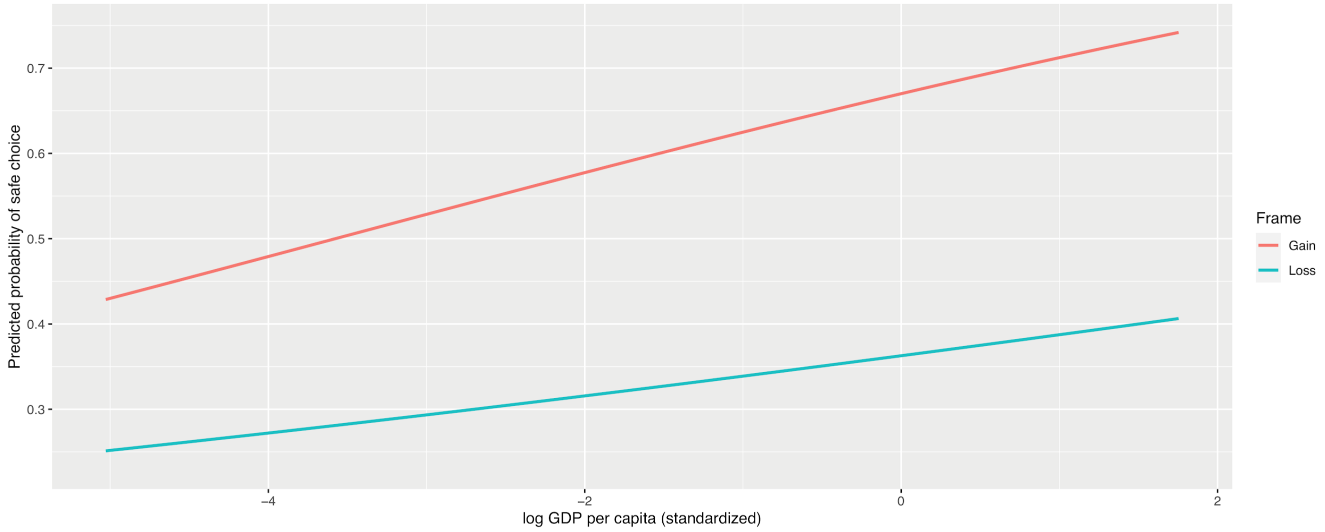

Figure S1.5

Relationship between the predicted probability of the safe choice and the frame moderated by compliance

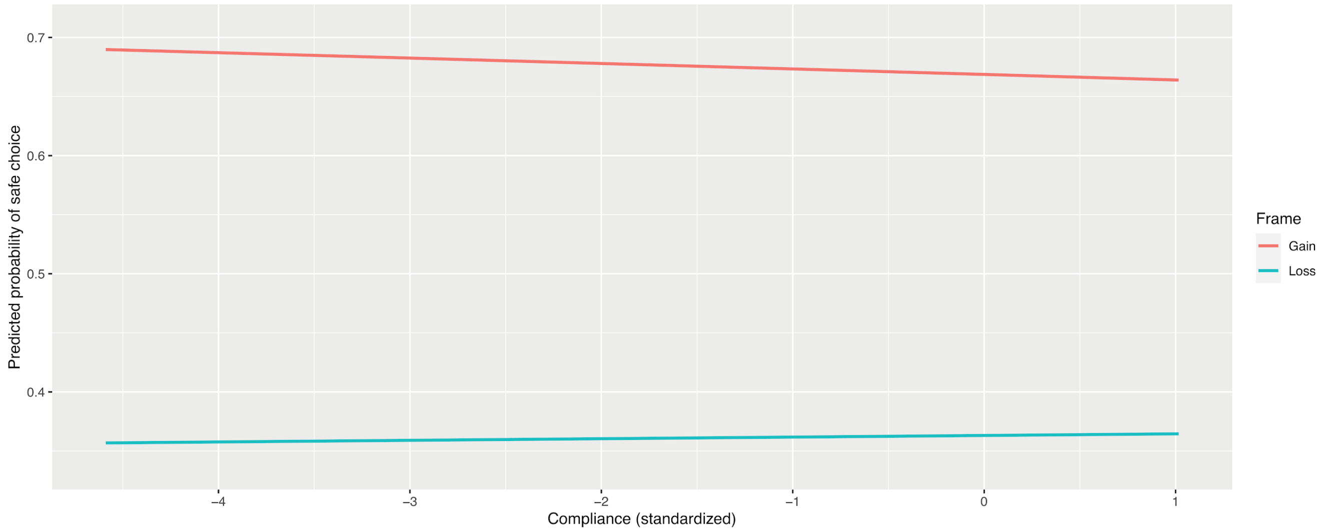

Supplement: S1 File — (PDF) [file pone.0257151.s001.pdf]
