# Supplementary material for: Replicating the Disease framing problem during the 2020 COVID-19 pandemic: A study of stress, worry, trust, and choice under risk
Source: PLoS One. 2021 Sep 10;16(9):e0257151. doi: 10.1371/journal.pone.0257151 (PMC8432807; doi:10.1371/journal.pone.0257151)

Replicating the Disease framing problem during the 2020 COVID-19 pandemic: A study of stress, worry, trust, and choice under risk

S2 File

Analyses Requested by Reviewers

Proportion of safe vs. risky choices in each framing condition in two studies

We examined how many participants selected the safe or risky option in each framing condition in our study (Table S2.1) and in the Many Labs study (Klein et al., 2014; Table S2.2).

Table S2.1

Disease Problem: Proportion of Safe and Risky Choices (Our study)

	Safe choice	Risky choice	Total
Gain	29,651 (66.90%)	14,692 (33.10%)	44,343 (50.29%)
Loss	15,919 (36.30%)	27,919 (63.70%)	43,838 (49.71%)
Total	45,570 (51.70%)	42,611 (48.3%)	88,181 (100%)

Table S2.2

Disease Problem: Proportion of Safe and Risky Choices (Klein et al., 2014)

	Safe choice	Risky choice	Total
Gain	1,974 (62.20%)	1,200 (37.80%)	3,174 (50.61%)
Loss	1,038 (33.50%)	2,059 (66.50%)	3,097 (49.39%)
Total	3,012 (48.00%)	3,259 (52.00%)	6,271 (100.00%)

Supplementary Analysis on Hypotheses 2a and 2b

Association between the distress level and safe choices in each framing condition

We examined the association between the distress level and safe choices in each framing condition (i.e., gain and loss). To examine the association, we performed two multilevel modeling analyses with the following model:

Choice ~ distress + (1 | country)

In the case of the gain condition, both frequentist and Bayesian multilevel modeling indicated that there was a significant positive association between the distress level and safe choices, B = .04, *SE* = .01, 95% Bayesian CI [.02 .06], β = .04, standardized *SE* = .01, *z* = 3.01, *p* < .01, *BF* = 1,999. However, the result from frequentist multilevel modeling demonstrated that the null hypothesis about absence of such an association could not be rejected in the loss condition, B = .01, *SE* = .01, β = .01, standardized *SE* = .01, *z* = 1.41, *p* = .08. Figure S2.1 displays the association in each condition:

Figure S2.1

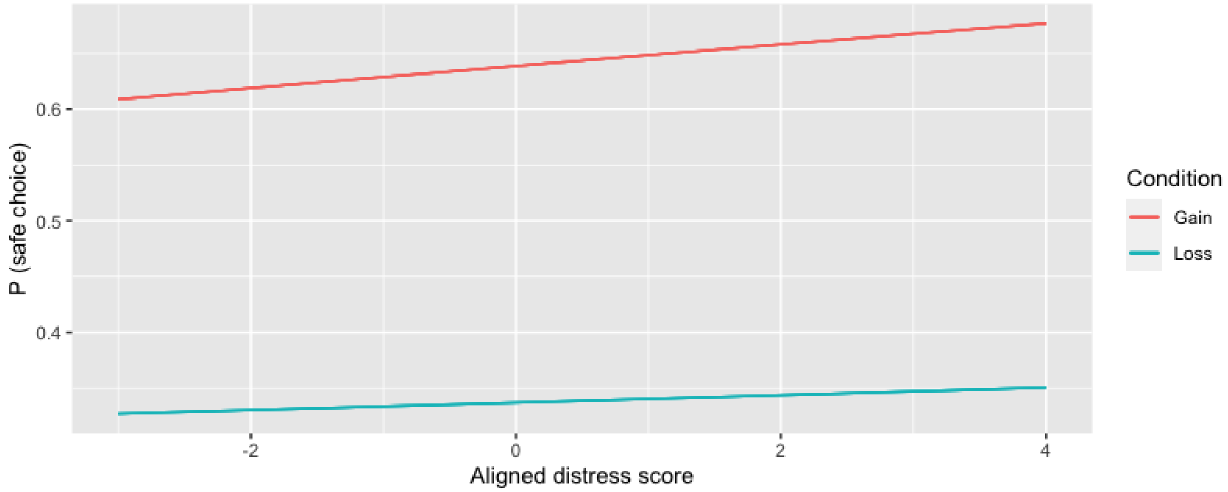

Comparison of the distress level during the first vs. second month during the survey period

To examine whether the participants were habituated to emotional experiences during the pandemic, we conducted additional multilevel modeling. In this analysis, we compared the distress level between participants who completed our survey during the first month of the survey period (April 25, 2020 or before) and those who completed the survey in the second month. To test the difference, we performed multilevel modeling with the following model:

Distress ~ month + (1 | country)

The result indicated that the distress level among participants who completed the survey in the second month was not significantly lower than that among those who completed the survey in the first month, $B = .00$, $SE = .01$, $t\ (88{,}020) = .38$, $p = .35$. Thus, the aforementioned potential habituation was not substantiated.

Relationship between participants' choices and COVID-19 deaths across different countries

We also examined to what extent COVID-19 deaths in each country was related to participants' safe choices and framing effect in each country. Because multilevel modeling allowed us to estimate differentiated intercepts and slopes across different countries, we were able to extract estimated random intercept and slope coefficients from the resultant multilevel model. For this purpose, the following model was examined

Choice ~ frame (1 | country)

The random intercepts (corresponding to the overall safe choices) and slopes (corresponding to the framing effect) were extracted from the model. Then, we tested correlation between these coefficients and log (cumulative COVID-19 deaths per million until 4/25/20)

across different countries. The random intercepts were significantly positively correlated with COVID-19 deaths, $r = .41$, $p < .001$, $BF = 12.75$; however, such correlation was not reported in the case of the framing effect, $r = .13$, $p = .38$. The results suggest that participants living in the hardest-hit countries were more likely to make safe choices, so they were more risk averse. However, the framing effect was not associated with COVID-19 deaths. Figure S2.2 illustrates the relationship between safe choices and log (cumulative COVID-19 deaths per million until 4/25/20):

Figure S2.2

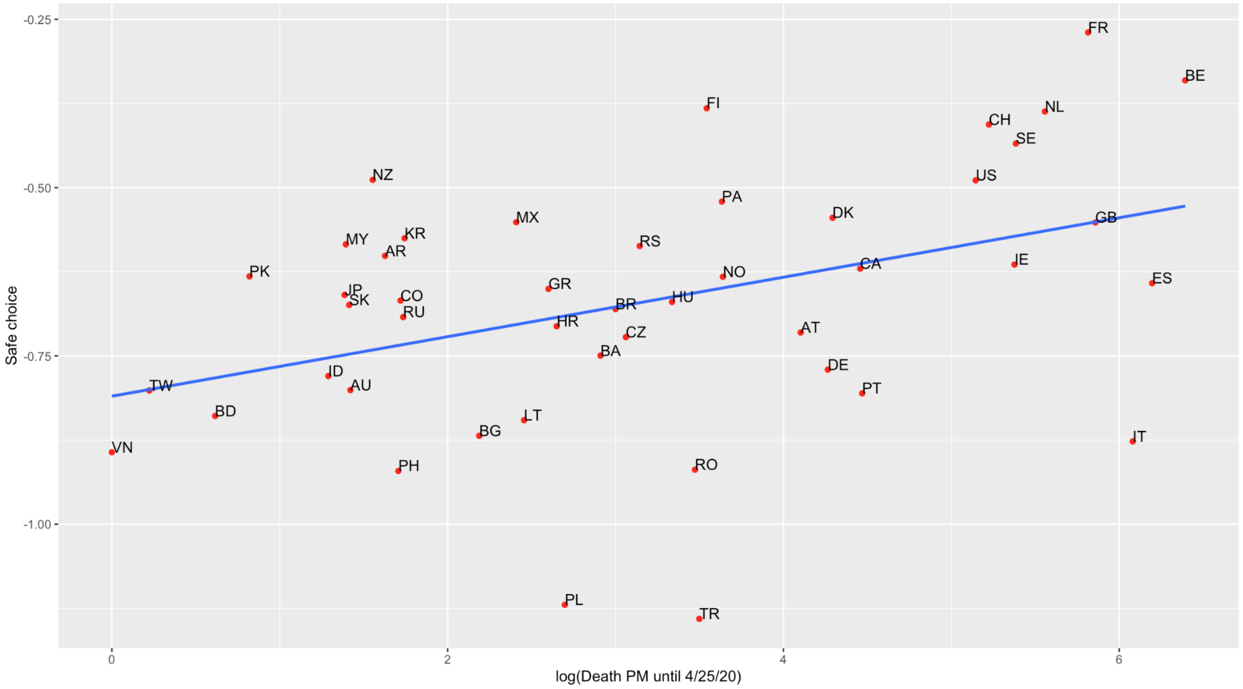

Supplement: S2 File — (PDF) [file pone.0257151.s002.pdf]
