# Supplementary material for: Replicating the Disease framing problem during the 2020 COVID-19 pandemic: A study of stress, worry, trust, and choice under risk
Source: PLoS One. 2021 Sep 10;16(9):e0257151. doi: 10.1371/journal.pone.0257151 (PMC8432807; doi:10.1371/journal.pone.0257151)
Supplement: Attachment — Submitted filename: PONE-D-21-06048_SB.pdf [file pone.0257151.s003.pdf]

This paper explores the framing effect in the Asian Disease Problem during the COVID-19 crisis. First, the paper investigates the framing effect across time. Second, the paper investigates the role of emotions in exacerbating or attenuating the framing effect. Third, the paper examines the association between macroeconomic measures and cross-country variation on choosing the safe option in the Disease problem and on the framing effect. Last, the authors investigate whether the interaction between compliance to preventative measures during the pandemic and the framing manipulation are associated with the frequency of choosing the safe option in the Disease problem. I see two main contributions in this paper:

1. It shows that the framing effect and self-reported stress were higher during the pandemic relative to the same measures elicited in previous years. Using individual-level analysis, it also shows that self-reported psychological distress enhances the framing effect.
2. It presents null results on the association between compliance behavior during the health crisis and the framing manipulation on the frequency of choosing the safe option or the framing effect.

Comments and Suggestions

1. There are a few papers that investigate emotions and the framing effect that have not been cited but can be used in the motivating paragraphs. See,
    a. Miu, A. C., & Crişan, L. G. (2011). Cognitive reappraisal reduces the susceptibility to the framing effect in economic decision making. *Personality and Individual Differences*, *51*(4), 478-482.
    b. Stanton, S. J., Reeck, C., Huettel, S. A., & LaBar, K. S. (2014). Effects of induced moods on economic choices. *Judgment and Decision Making*, *9*(2), 167.
2. Anger and hostility are conflated with distrust. Trust is a belief whereas the others are not. These terms should be separated and not used interchangeably, unless the survey measure direct links between them. These terms are conflated in the motivation and discussion sections.
3. Instead of comparing stress in 2020 to that in 2009, have you considered splitting the sample into responses collected in the first month versus that in the second month? If the stress response is lower during the second month as emotional experiences habituated, you may have cleaner comparison than the more than a decade-long comparison. This may also serve as a robustness check for the reported results. If habituation does exist, then similar analysis can be applied for the framing effect.
4. How were countries different in terms of risky choices and the framing effects? It would be reasonable to examine whether countries with greater COVID-19 deaths had an exacerbated framing effect. The paper mentions that it's beyond the scope of this paper, but it seems quite reasonable to include it here since you devoted a section discussing cross-country variability.
5. Have you considered investigating whether the distress measure was associated with increased risk aversion within each frame condition separately or not? This sub-sample analysis may be interesting as the distress-frame interaction effect may be present if, for example, the frequency of safe choices increases in both frames with the gain domain

increasing more vigorously or the frequency of safe choices increases in the gain domain while that of the loss domain falling.

6. To investigate potential selection bias, did you compare observed characteristics such as gender or age between included and excluded participants? If not, then you need to do so and please report.
7. You need to do an analysis that test whether familiarity with the framing effect mattered OR cut all mention of collecting that data and the associated section. It isn't reasonable to claim that researchers should control for familiarity if it doesn't change participant responses.
8. As it stands, the conclusions section is weak. You might think about how the main results can be used by authorities and researchers when communicating during a crisis.
9. This paper needs substantial editing before it can be published. You should consider hiring a professional science editor. In particular, the number of words can easily be cut by 15% by just eliminating wordiness and redundancy. This will result in a paper that is much easier to read. Here are a few specific suggestions, but this is not a comprehensive list:
    a. The introduction and discussion sections need to be shortened as there is multiple overlap.
    b. Edit the abstract so that it has only the most important results. For example, the sentence about GDP isn't central to your paper (the title suggests it is about stress and worry, not the economy.) The abstract will sound stronger if there are fewer results that are more closely tied together.
    c. This is science writing and the goal is not to evoke the reader's emotions, so please avoid editorializing and using extra adjectives. For example, the word "eerily" in the first sentence. Line 90-92 doesn't need the parenthetical remark (that is missing a parenthesis) about omitting "Asian".
    d. Rephrase the questions in the first paragraph so they sound more like "aims." While you are at it, the sentence that starts with "Answering…" doesn't really tell the reader anything useful. This is a good example of where editing can help.
    e. The "Practical Applications" section goes into the discussion at the end. It is awkward to have it after the hypotheses have already been presented.
    f. Please resist the temptation to start many sentences in the literature review section with the author's names (e.g. Smith found this…. Jones looked at Smith's work and found this….). Keep the focus on the results – interested readers can look at the references section to see whose work it is.
    g. Lines 141-144 contain redundant sentences.
    h. In page 24: line 515, an awkward sentence about BF: "…becomes more favor a null hypothesis."
    i. Lines 525-6. You can cut the sentence that starts with "Accordingly…."
    j. Lines 701-703 can be made a single, more succinct sentence.
    k. Lines 762, cut the sentence that starts "We thus…"
    l. Line 774, cut the phrase "Assuming our findings reflect reality" and rephrase the question as a statement.

m. In page 36: line 785, an awkward sentence about findings: "…findings present both support but also challenges that the ATF has to address."
n. In the discussion section, consider omitting ambiguous statements such as "assuming our findings reflect reality" and "for some reason."
o. Don't lead the conclusions section with limitations.
p. Line 966 I would replace "particularly in times like the COVID-19 pandemic which has introduced a new element of risk and uncertainty to the lives of people around the world." with "during a crisis."
