# Supplementary material for: Replicating the Disease framing problem during the 2020 COVID-19 pandemic: A study of stress, worry, trust, and choice under risk
Source: PLoS One. 2021 Sep 10;16(9):e0257151. doi: 10.1371/journal.pone.0257151 (PMC8432807; doi:10.1371/journal.pone.0257151)
Supplement: Attachment — Submitted filename: Response to Reviewers_Fin.docx [file pone.0257151.s004.docx]

Response to Reviewers

Dear Dr. Aimone, dear reviewers,

Thank you very much for your feedback. We highly appreciate the time and effort you dedicated to providing insightful comments and valuable suggestions to our submitted manuscript. We were excited by the opportunity to revise our manuscript. We have incorporated most of your suggestions and believe we have thereby considerably improved our manuscript. In what follows, we first present a general summary of the major changes we made from our previous submission and then reply to each individual point.

# General Summary

Following the suggestions of all three reviews, our primary effort in the revised version was to improve readability of the paper by making it clearer, more concise, and more coherent. We put much effort to eliminate wordiness and redundancy. We also restructured parts of the paper in a more logical order and improved connections between individual ideas. The resulting paper is 3,300 words shorter and, we believe, much better focused on its research questions.

In the introduction, we removed or shortened non-essential parts. In the same time, we tried to make it clear that our primary research goal is to test predictions from the appraisal-tendency framework. Following your suggestions, we moved sections related to cross-national variability and compliance before the hypotheses. We also added a section reporting our preregistered exploratory analyses.

We restructured or removed parts of the Materials & Methods section to make it flow more logically. In response to your request, we also added information regarding potential selection bias. We described our analytic approach more clearly (in particular, our model selection strategy), which also allowed us to report the results more concisely.

By also removing wordiness redundancy, we were able to keep our Results section at roughly the same size as in the original version despite the new analyses included in response to the reviewers’ requests. We also added a new supplement (Supplement 2) that provides more detail on the new analyses. Finally, we thoroughly rewrote the Discussion to make its main points clearer and more visible.

We are grateful for every point or concern you have raised. We believe that each one has helped us tremendously in improving our manuscript. Please find our point-by-point response to your points below.

# Editor

## 1. Comparing new data to old data: The influence of unobserved factors

First and foremost the major motivation and hypotheses appear to be comparing your new data to that of data collected in a different paper in 2014. While I think it is safe to conclude/assume that distress is different in these two data sets, there is quite substantially more things that have occurred in the gap period that could plausibly affect differences as well. I do not see a way to remove the plausibility of these other unobserved factors being responsible for observed differences. While any comparisons within your new data set do not have that problem, any discussion of differences to a pre-covid era data set would need to provide some form of additional identification mechanism. I do not see this as feasible. That said, the data set you do have is rich, but as written the paper seems to rest too much on a comparison with no identification mechanism.

**Response:** Thank you for raising this point. We agree with the criticism regarding the comparison between the two datasets. Contrary to the impression we might leave the reader with, our original focus was primarily on evidence within our dataset, for reasons mentioned above: it is rich enough and does not suffer from limitations due to unobserved factors. Accordingly, we have six hypotheses related to analyses within our data versus only two related to analyses between our data and the reference dataset.

Your criticism helped us realize that we have failed to clearly convey our intentions in the original version. In the revised introduction, we explicitly state that Hypotheses 2a–d provide a stronger and more direct test, than Hypotheses 1a–b, of ATF’s predictions regarding the associations between stress, safe choices, and the framing effect (ln. 137–142). Similarly, in the Discussion, we state that the “positive, but indirect evidence for an association between stress and risk aversion is qualified, however, by the non-significant results of testing the association directly within our study” (ln. 581–583). In summing up hypotheses related to risk avoidance, we also emphasized on null findings within our data rather than on the positive finding from the comparison between the two datasets (ln. 637–639). Overall, we devoted much more space to discussing results regarding Hypotheses 2a-f concerning analyses within our data than Hypotheses 1a-b concerning comparisons between datasets (see Discussion, Section “Testing the Appraisal-Tendency Framework’s Predictions”).

We still think that, despite alternative explanations and possible hidden moderators, Hypotheses 1a–b are worth testing and the corresponding analyses provide important information. We are aware that by putting Hypotheses 1a–b at the start of the list we seem to emphasize on these but we refrained from rearranging the hypotheses to keep the enumeration as preregistered.

## 2. A lot of data exclusions

Second, you lost a lot of data to exclusions. It seems you lost about half of your data (173k down to 88k). This seems excessive and worrisome. How can we be confident that your remaining data is still a representative data set? Could any of the factors that contributed to individuals being excluded also be related to decision of interest behavior? Speed, completeness of answering questions, etc. could presumably be associated with dedication, honesty, concern for others, etc. that would presumably be related to variables of interest. Your data set is also very lopsided on gender balance. Was gender balance before exclusions were implemented more or less or similarly balanced? Does analysis look similar if you engage a weaker set of exclusion criteria? Such concerns need to be addressed.

**Response:** We agree that 173,426 down to 88,181 seems like a lot of exclusions. However, as stated in our manuscript, our starting point was the cleaned dataset (*N* = 125,306) pre-processed by Yamada et al. (2021) following preregistered criteria (i.e, participants of at least 18 years of age who gave informed consent). Using the large number of participants who have not met those criteria would obviously be questionable for ethical reasons.

All further exclusions we made were preregistered and in line with similar preprocessing using our data (Lieberoth et al., 2021). The largest dropout (*n* = 31,171 or 25% of the cleaned dataset) is produced by responses that include missing values on some of the variables of interest. We have included this criterion for the purpose of measurement invariance and alignment which require responses on all individual items. We also excluded responses that took less than 2 min 12 sec, which is one tenth of the estimated time to take the survey (*n* = 1,288 or 1%). Those incredibly fast responses are most likely produced by inattentive responding and are thus not valid responses. Consequently, they are routinely excluded prior to analyses. We excluded observations from countries with less than 100 participants (*n* = 1,211, 1%) for the purpose of measurement invariance and alignment. Finally, we excluded multivariate outliers (*n* = 3,455, 3%), which is also a routine practice to detect random or carelessly invalid responses (Curran, 2016; Dupuis et al., 2019). Since online data collection lacks the procedural control of laboratory studies, it is not surprising that the dropout rate is larger than in datasets collected in person, with an average of about 30 percent (Galesic, 2006). Incidentally, we also have roughly a 30 percent overall dropout rate. The rate of our exclusions is thus not unusual and we believe that excluding those observations contributed to getting high-quality data from our large-scale survey.

In response to your point, we also examined the gender balance before and after exclusions. We found that the potential selection bias is not a significant issue in the present study. (ln. 281–289).

## 3. Different degrees of exposure to the pandemic

Third, touched on in the reports, is the idea of the degree of pandemic exposure differences between the different countries in the data set. Covid had not spread as far in late March as later in the data period. You should control for country level exposure. One reviewer suggested Covid Deaths, but you may want to explore something like Cases per 100,000 individuals or some other measure that adjusts for country population.

**Response:** We appreciate your remark. In response, we extracted the overall safe choices and framing effect in each country from the estimated random intercepts and slopes in the estimated multilevel model. Then, we examined the correlation between these two indicators and log (cumulative COVID-19 deaths per million until 4/25/20, the midpoint of the survey period). The results indicated that the overall safe choices were positively correlated with COVID-19 deaths. It suggests that participants living in hardest-hit countries were most likely to be risk averse. However, such a relationship was not found in the case of the framing effect. Further details regarding this additional analysis are now added to the main text (ln. 742–475) and Supplement 2. We believe that these additional analyses do not necessarily qualify or are directly relevant to our main findings, as we have already modeled cross-country differences as random slopes and intercepts within our multilevel models.

## 4. Length

Finally the paper is very long and cumbersome. While the topic/motivation seems concise the implementation is complicated.

**Response:** Thank you for your comment, also shared by the reviewers. In the revised version, we aimed to keep the paper as focused and concise as possible. We considerably reduced the size of the Introduction and Discussion sections by removing unnecessary details and redundant information. We also restructured the Introduction which, we believe, made it more to the point. Despite including new analyses requested by the reviewers, we were able to keep the Method and Results sections at roughly the same length. We also tried to use clearer and more concise phrasing throughout. We hope that our efforts resulted in a more focused and reader-friendly paper.

# Reviewer #1

See attached referee report for suggestions for additional data analysis and editing the paper. This main result about framing is very interesting. I suggest that the authors think about the implications of this result for public health officials, and include comments in the conclusions.

This paper explores the framing effect in the Asian Disease Problem during the COVID-19 crisis. First, the paper investigates the framing effect across time. Second, the paper investigates the role of emotions in exacerbating or attenuating the framing effect. Third, the paper examines the association between macroeconomic measures and cross-country variation on choosing the safe option in the Disease problem and on the framing effect. Last, the authors investigate whether the interaction between compliance to preventative measures during the pandemic and the framing manipulation are associated with the frequency of choosing the safe option in the Disease problem. I see two main contributions in this paper:

1. It shows that the framing effect and self-reported stress were higher during the pandemic relative to the same measures elicited in previous years. Using individual-level analysis, it also shows that self-reported psychological distress enhances the framing effect.

2. It presents null results on the association between compliance behavior during the health crisis and the framing manipulation on the frequency of choosing the safe option or the framing effect.

**Comments and Suggestions**

## 1. Relevant References

1. There are a few papers that investigate emotions and the framing effect that have not been cited but can be used in the motivating paragraphs. See,

a. Miu, A. C., & Crişan, L. G. (2011). Cognitive reappraisal reduces the susceptibility to the framing effect in economic decision making. Personality and Individual Differences, 51(4), 478-482.

b. Stanton, S. J., Reeck, C., Huettel, S. A., & LaBar, K. S. (2014). Effects of induced moods on economic choices. Judgment and Decision Making, 9(2), 167.

**Response:** Thank you for these suggestions. We have not included Miu and Crişan because their recruitment strategy (small samples of individuals highly skilled in a specific reappraisal strategy) and their lack of a control group limits the generalizability of their results. We have, however, incorporated Stanton et al., in the updated review of the role of emotions in framing (ln. 90–103).

## 2. Anger and hostility conflated with distrust

2. Anger and hostility are conflated with distrust. Trust is a belief whereas the others are not. These terms should be separated and not used interchangeably, unless the survey measure direct links between them. These terms are conflated in the motivation and discussion sections.

**Response:** Thank you for this important remark. In the revised paper, we thoroughly inspected each instance of mentioning trust/distrust and took special care not to conflate them with anger and hostility, and with emotions in general. In the introduction, we also cited evidence that distrust and anger are interrelated by virtue of their shared emotional tone and cognitive appraisals (ln. 144–155), thus providing an improved rationale for our Hypotheses 2e–f.

## 3. Split the Sample & Compare 1st and second Month

3. Instead of comparing stress in 2020 to that in 2009, have you considered splitting the sample into responses collected in the first month versus that in the second month? If the stress response is lower during the second month as emotional experiences habituated, you may have cleaner comparison than the more than a decade-long comparison. This may also serve as a robustness check for the reported results. If habituation does exist, then similar analysis can be applied for the framing effect.

**Response:** We appreciate your comment regarding the possible change in the stress level during the survey period due to a habituation. Following your comment, we conducted additional multilevel modeling comparing the stress level between participants who completed the survey in the first versus the second month. The effect of the month of participation was not significant. There is thus no evidence for habituation during the survey period. We reported the analysis briefly in the manuscript (ln. 470–472) and, in more details, in Supplement 2.

## 4. Details about cross-national differences

4. How were countries different in terms of risky choices and the framing effects? It would be reasonable to examine whether countries with greater COVID-19 deaths had an exacerbated framing effect. The paper mentions that it’s beyond the scope of this paper, but it seems quite reasonable to include it here since you devoted a section discussing cross-country variability.

**Response:** Thank you for your suggestion. As an instance of such follow-up analysis, and following the Editor’s and Reviewer 2’s suggestions, we extracted the overall safe choices and framing effect in each country from the estimated random intercepts and slopes in the multilevel model. We the correlated these two indicators to the log (cumulative COVID-19 deaths per million until 4/25/20, the midpoint of the survey period). The overall safe choices were positively correlated with COVID-19 deaths, suggesting that participants living in hardest-hit countries were most likely to be risk aversive. However, such a relationship was not found in the case of the framing effect. We reported the analysis briefly in the manuscript (ln. 472–475) and, in more details, in Supplement 2

## 5. Explore association between distress and risk aversion within each frame

5. Have you considered investigating whether the distress measure was associated with increased risk aversion within each frame condition separately or not? This sub-sample analysis may be interesting as the distress-frame interaction effect may be present if, for example, the frequency of safe choices increases in both frames with the gain domain increasing more vigorously or the frequency of safe choices increases in the gain domain while that of the loss domain falling.

**Response:** Thanks a lot for your suggestion. We performed the analyses and found that the distress level was positively associated with safe choices only in the gain condition, not in the loss condition. We reported the analysis briefly in the manuscript (ln. 467–470) and, in more details, in Supplement 2.

## 6. Compare gender & age of included vs. excluded to test for selection bias

6. To investigate potential selection bias, did you compare observed characteristics such as gender or age between included and excluded participants? If not, then you need to do so and please report.

**Response:** We appreciate your comment on the potential selection bias. We compared the gender and age of participants who were included in versus excluded from the pre-processed dataset. The analysis showed that selection bias would not be a significant issue in the present study (ln. 281–289).

## 7. Effect of familiarity on framing

7. You need to do an analysis that test whether familiarity with the framing effect mattered OR cut all mention of collecting that data and the associated section. It isn’t reasonable to claim that researchers should control for familiarity if it doesn’t change participant responses.

**Response:** Thank you for your point. In fact, we have already included such an analysis in the original version (see lines 684–699). In the revised version, we tried to make it more easily noticeable by 1) announcing it in the Introduction, Exploratory Analysis section (ln. 229–231), 2) improving the report in the Results section (ln. 543–555), and 3) elaborating on the findings in the Discussion (ln. 707–716). In the revised version, we also used a consistent order of the exploratory analyses across different sections, with analyses related to familiarity always coming last. We hope that we thus made the information about familiarity, as well as the other exploratory analyses, more visible to the reader.

## 8. Weak Conclusions Section

8. As it stands, the conclusions section is weak. You might think about how the main results can be used by authorities and researchers when communicating during a crisis.

**Response:** Thank you for this suggestion. Our main positive finding, that distress and concerns moderate the framing effect, relates to predictions from the appraisal-tendency framework. Its practical relevance is not immediately apparent. Our exploratory analysis of framing and compliance might be of more practical use as it suggests that the framing manipulation has limited effectiveness as a tool to promote compliant behavior. In the discussion, we consider convergent evidence beyond our study showing that stable individual characteristics such as risk tolerance, patience, social responsibility, etc. might be more powerful predictors of compliant behavior than subtle context manipulations such as the way a message is framed. We briefly reiterate this idea in the conclusion. However, our evidence is only exploratory and is inconclusive since it is based on null findings. Therefore, we refrained from further elaborating on the issue. We believe that our conclusions already imply that authorities and researchers should be cautious when expecting framing manipulations to make a considerable difference in public behavior.

## 9. Editing Needed

9. This paper needs substantial editing before it can be published. You should consider hiring a professional science editor. In particular, the number of words can easily be cut by 15% by just eliminating wordiness and redundancy. This will result in a paper that is much easier to read. Here are a few specific suggestions, but this is not a comprehensive list:

a. The introduction and discussion sections need to be shortened as there is multiple

overlap.

b. Edit the abstract so that it has only the most important results. For example, the sentence about GDP isn’t central to your paper (the title suggests it is about stress and worry, not the economy.) The abstract will sound stronger if there are fewer results that are more closely tied together.

c. This is science writing and the goal is not to evoke the reader’s emotions, so please avoid editorializing and using extra adjectives. For example, the word “eerily” in the first sentence. Line 90-92 doesn’t need the parenthetical remark (that is missing a parenthesis) about omitting “Asian”.

d. Rephrase the questions in the first paragraph so they sound more like “aims.” While you are at it, the sentence that starts with “Answering...” doesn’t really tell the reader anything useful. This is a good example of where editing can help.

e. The “Practical Applications” section goes into the discussion at the end. It is awkward to have it after the hypotheses have already been presented.

f. Please resist the temptation to start many sentences in the literature review section with the author’s names (e.g. Smith found this.... Jones looked at Smith’s work and found this....). Keep the focus on the results – interested readers can look at the references section to see whose work it is.

g. Lines 141-144 contain redundant sentences.

h. In page 24: line 515, an awkward sentence about BF: “...becomes more favor a null hypothesis.”

i. Lines 525-6. You can cut the sentence that starts with “Accordingly....”

j. Lines 701-703 can be made a single, more succinct sentence.

k. Lines 762, cut the sentence that starts “We thus...”

l. Line 774, cut the phrase “Assuming our findings reflect reality” and rephrase the question as a statement.

m. In page 36: line 785, an awkward sentence about findings: “...findings present both support but also challenges that the ATF has to address.”

n. In the discussion section, consider omitting ambiguous statements such as “assuming our findings reflect reality” and “for some reason.”

o. Don’t lead the conclusions section with limitations.

p. Line 966 I would replace “particularly in times like the COVID-19 pandemic which has introduced a new element of risk and uncertainty to the lives of people around the world.” with “during a crisis.”

**Response:** We are grateful for your careful reading and relevant suggestions. We have implemented *all* of them into our revised paper. More generally, we put much effort to eliminate wordiness and redundancy, as well as information not directly relevant to our research aims. We hope that the result is a more focused and reader-friendly paper.

# Reviewer #2

*** General comments

## 1. General Comments

Generally, the paper focuses on an interesting topic, it is well-written and the statistical analyses are conducted with great care.

A main problem is that the paper focuses on many different dimensions and analyses. Overall, it is way too long and needs to be shortened.

In this respect, to increase clarity, the authors should also try to better connect and combine the different fields of the research questions they ask (e.g., the effect of emotions on the DP paradigm and the inter-country effects could be bundled).

**Response**: Thank you for your feedback. Making the paper clearer and more coherent was our primary goal during revising. However, we originally derived the hypotheses related to emotions separately from the hypotheses related to cross-national differences, drawing on quite different sources in each case. There might be limits to which these separate parts can be connected post hoc, although there might also be links that we are missing. We hope that the complexity added by investigating several separate topics is handled better in our shortened and better-structured revised paper.

Below, I present may comments, which are ordered by section.

*** Introduction

## 2. New References

- The authors provide interesting examples for the application to pandemic management and compliance with the healthcare guidelines. Here, it would be important that they cite the recent literature studying these topices (Campos-Mercade et al., 2021; Müller and Rau, 2021).

- In the context of associations between risky choice and social distancing Müller and Rau (2021) also provide survey evidence of the COVID-19 pandemic. In Müller and Rau (2021) a significantly negative relation was found between risk tolerance and compliance to healthcare guidelines.

**Response:** Thank you for these suggestions. We are now citing both references in the Introduction (ln. 174–175) as well as in the Discussion (ln. 559–560).

*** Competing Accounts for the Framing Effect

## 3. Sloppy explanation of prospect theory

- on p. 6 the Prospect Theory explanation is a bit sloppy. The authors state: "Consequently, the guaranteed gain of 200 people being saved is evaluated as a greater gain than a one-third chance of saving 600 lives.."

=> The reason why the first gamble leads to a higher utility in the "prospect theory world" is the reference point (i.e., the sure gain of 200 people in the first gamble). Comparing this reference point to the second gamble, it may be that people enter the loss domain, i.e., when no-one is saved, we have a loss of -200.). In combination with loss aversion (i.e., losses loom larger than gains) the second gamble leads to a lower prospect theory utility.

=> Similarly, in the second example, peoplke's pronounced sensitity to losses (i.e., loss aversion) is the reason why pepople in the domain of losses try to avoid sure losses and start to gamble.

This shold be addressed, i.e., the authors should improve the explanation of the gamble choices according to the idea of Prospect Theory.

**Response:** We agree that our description of prospect theory was incomplete. However, we respectfully disagree with Reviewer #2’s interpretation of the reference point. For instance, Reviewer #2 has proposed that the sure gain of 200 people in the gain frame is the reference point. By contrast, Kahneman and Tversky (1984, p. 343) have stated that “The formulation of Problem 1 implicitly adopts as a reference point a state of affairs in which the disease is allowed to take its toll of 600 lives.” That is, the reference point is the loss of 600, rather than the gain of 200. Similarly, the loss frame “assumes a reference state in which no one dies of the disease.” (Kahneman & Tversky, 1984, p. 343). Also, loss aversion, while very important in prospect theory, is not directly involved in explaining the framing effect in particular, which can be accounted for by the two other founding principles of prospect theory: reference point and diminished sensitivity.

We are happy to discuss this point further. However, during the revision we realized that description of prospect theory is tangential to our study. Therefore, following our strategy to cut unnecessary details, we decided to shorten rather than expand it, as we also did for other accounts of the framing effect that are not directly relevant to our study. We hope that this led to a better-focused paper.

*** Predictions setting

## 4. Present Klein et al. (2014) in more details

- The paper does a good job in summarizing the literature and integrating the current study in the literature. Since, the current study compares its finding to the "Many Labs replication" of Klein et al. (2014) it would be helpful for the reader, if this study (and its circumstances) could be better explained and introduced. Currently, the author solely write: "..presumably run under less stressing circumstances."

**Response:** Thank you for this suggestion. In response, we have added relevant details on the Many Labs study (Klein et al., 2014), ln. 126–136.

## 5. H1b imprecise

- H1b: The authors write: "the framing effect on the disease problem would be higher than typical" This is too imprecise. What do the authors mean with "higher"? The relative change in proportions of the safe choices? More detailed explanation is needed.

**Response:** Thank you for this comment. We kept the phrasing of H1b as preregistered but now have also clarified that we mean “lаrger” in terms of Cohen’s *d* (ln. 204), which was our original plan. We have not made specific predictions regarding the change of proportions. Since Cohen’s is an indicator of effect size, we tested H1b by calculating and comparing the respective estimates for the Many Labs and our study. We believe that this is a direct test of our H1b.

## 6. H2a-d not needed

- I doubt that hypotheses 2a-2d are needed. As I understood it correctly, these hypotheses are related to the role of emotions on the framing effect during the pandemic. This is actually, what is also tested in hypotheses 1a and 1b.

Put differently, the reason why there should be differences as compared to Klein et al. (2014) is motivated by the changed emotions during the pandemic and their impact on frmaing (which is motivated in hypotheses 2a-2d).

I would recommend to drop hypotheses 2a-2d. Instead, hypotheses 1a and 1b should be more precisely motivated by the changes in emotions during the pandemic (see hypotheses 2a-2d). Hypotheses 1a and 1b could be accompanied by hypotheses 2e and 2f, which could turn to hypotheses 2a) and 2b).

**Response:** Thank you for this comment. We agree that Hypotheses 1a–b and 2a–d are similar in that they test predictions derived from the appraisal-tendency framework (ATF) regarding the role of emotions in risky choices and the framing effect. Yet, the two sets of hypotheses are also fundamentally different from one another. In particular, while Hypotheses 1a–b test ATF’s predictions by comparing our data with the reference data by the Many Labs (Klein et al., 2014), Hypotheses 2a–d test those predictions entirely *within* our data. We thus believe that, contrary to your suggestion, hypotheses 2a–d are in fact more important than 1a–b because the evidence they provide is not subject to alternative explanations and hidden variables. In the revised version, we tried to make this point clearer both in the introduction, ln. 137–143, and the discussion, ln. 581–585 (see also our response to Editor’s Point #1).

*** Sample

## 7. Female-dominated Sample

- It is conspicuous that the majority of the participants in the sample is female (72.5%). Regarding common gender differences in risk taking (i.e., women are more risk averse than men) it is possible that this composition towards such an extreme share of women may have led to an upward bias of safe choices. In this respect, it would be important to compare and discuss the gender composition in Klein et al. (2014). Ideally, an overview table with the sample characteristics of Klein et al. (2014) should be presented and compared to the sample of the current study.

- How do the data look like, if the authors weight it in terms of the gender composition?

**Response:** Thank you very much for your comment. We admit the fact that the majority (73.37%; only among who reported their gender) of the participants were female. However, the majority of participants in Klein et al. (2014) were also female (69.77%). Due to the large sample sizes, the difference in these two proportions is statistically significant but the effect size of the difference in terms of the odds ratio is negligible, 1.19. Hence, we assumed that gender could not be a significant issue while comparing our study with Klein et al. (2014). The analysis is reported in the revised version, ln. 294–298.

*** Results

## 8. Check US stress levels between 2010 and 2019

- Regarding the robustness check of the mean PSS-10 score among Americans in the current study, could it be that the US stress levels generally rose since 2009 (e.g. for different reasons)? It would be interesting to focus compare the PSS-10 levels between 2009 and the mean of the period between 2010 and 2019. If there is no difference this would be convincing.

**Response:** Thank you for your comment. Although we could not find any additional relevant studies that addressed the change in PSS-10 among Americans during the period of 2009-2019, we found the American Psychological Association’s annual reports, the Stress in America reports for additional information (American Psychological Association, 2014, 2019). According to the reports, the overall stress level among Americans decreased during that period. This survey outcome may suggest that the significantly greater stress level in 2020 reported in our study compared with that in 2009 was not due to an increasing trend but perhaps to the 2020 pandemic.

## 9. Report descriptive statistics

Regarding the test of H1a:

- It would be interesting, if the authors could report some descriptive statistics before turning to the regression analyses That is, what is the mean share of safe choices in each frame in the current study and how did this look like in the reference study?

**Response:** We appreciate your suggestion. We have added the tables to Supplement 2.

## 10. Does power affect the difference in effect size?

Regarding the test of H1b:

- If the idea of the analysis of H1b solely relies on showing a difference in the effect size, I wonder to what extent this result is driven by the difference in the power of the current study. This should be made more clear.

**Response:** Unlike statistical significance, effect size is independent from sample size. For instance, Cohen’s *d* is defined as the difference between two means divided by the standard deviation. Neither the means nor the standard deviation are related to sample size. More generally, increasing the sample size leads to an increase of statistical power, that is, an increased likelihood to detect a statistically significant effect of a particular size. However, it does not affect the size of the effect itself. Thus, our huge sample size could not have affected the effect sizes in the present study. The independence between an effect size and sample size (or power) is described in details by Sullivan and Feinn (2012).

## 11. By-gender comparisons of the US samples

Regarding the idea of the sub sample ronustness checks:

- As a robustness check (to harmonize the samples), the authors test H1b by solely focusing on the US samples. How does such a sub sample comparison look like when comparing the male and female date across both studies?

**Response:** Thank you for your suggestion. We ran additional Bayesian multilevel modeling and found that the framing effect was significantly greater in our study compared with the Many Labs [(Klein et al., 2014)](https://www.zotero.org/google-docs/?urr8Su) among female participants but not male participants. We briefly report this in the main manuscript (ln. 452–456).

In both our study and the Many Labs the majority of were female (73.37% and 69.77%, respectively). Therefore, the two datasets are comparable in terms of the generalizability of the findings across genders. We thus hold that the overall framing effect in our study was significantly greater than that in Klein et al. (2014). One more point to consider is that the number of male participants in Klein et al. (2014) was relatively very small, *n* = 889 (vs. 23,205 in our study). Therefore, the comparison of male participants across the two studies might not as generalizable as the comparison of female participants. Hence, we believe that by-gender comparison between the Many Labs and our study does not add crucially important information regarding robustness but we report it anyway.

## 12. Interpretation of the non-significant relation between safe choices and compliance:

- An explanation may be that the compliance question was very broad and did not directly contain connections to risky behavior during the crisis (i.e., staying at hoome, avoidance of large crowds, eic). Instead, the way how the question was addressed may also refer to a more general behvior similar to cooperation, or social preferences, which is of different nature than risky decisions. This should be addressed/discussed.

**Response:** Thank you for this suggestion. We incorporated it in discussing the non-significant association between safe choices and compliance (ln. 666–669).

Interpretation of the increased preference for safe choices in the pandemic:

## 13. Evidence that risk aversion increases during the pandemic

- Bu et al. (2020) find evidence for increased (temporarily) risk averion of subjects in Wuhan. They argue that this is related to their exposure to the crisis. They argue that higher exposure to the criss (an higher levels of perceived stress) increase risk aversion. This argument of increased risk aversion (Bu et al., 2020) during the pandemic could also be used for the authors' argument that the induced framing may be less effective, if the current distress level is already quite high.

**Response:** Thank you for this suggestion. In fact, we found that the non-significant association between level of stress and safe choices (not the framing effect) is unlikely to be accounted for by stress levels being too high: we found evidence for neither a ceiling effect nor a reduced variation of stress and concerns scores. We did, however, incorporate Bu et al. (2020) in our paper as it provides evidence consistent with our Hypotheses 1a regarding global increase in stress and risk aversion during the pandemic (ln. 577–581).

## 14. Association between risk aversion and exposure to the crisis within our data

- What do the data tell for countries with a higher exposure to the crisis? For instance, this could be proxied bny focusing on the infection or death rates of the countries.

**Response:** Thank you for your suggestion. To test it, we extracted the overall safe choices and framing effect in each country from the random intercepts and slopes in the estimated multilevel model. Then, we examined the correlation between those two indicators and log (cumulative COVID-19 deaths per million until 4/25/20, the midpoint of the survey period). Safe choices per country were positively correlated with COVID-19 deaths, suggesting that participants living in hardest-hit countries were more risk averse. However, such a relationship was not found in the case of the framing effect. We briefly reported this finding in the main text (ln. 472–475) and, in more detail, in Supplement 2.

## 15. Details on country-specific differences

- The authors argue that they could not infer country specific differences in the present study (I am also aware that this may be exhausting), but nevertheless I believ that this kind of heterogeniety is really important to better explain the outcomes. Therfore, it would be important to learn more about the country-specific effects, related to the COVID-19 crisis.

**Response:** We appreciate your comment and agree that country-specific effects are interesting and valuable. For instance, the association between safe choices and COVID-19 deaths discussed above suggests that our open data can be subject to promising exploratory analyses. Yet, we refrained from delving into further such analyses, as these would go against our primary goal to make our revised paper more concised and focused.

More generally, a thorough study at the country level should be both theoretically grounded and carefully designed to measure the covariates of interest while controlling for a host of possible confounds. Consequently, country-level analyses were originally beyond the scope of our study. Instead, we statistically controlled for heterogeneity by adding random intercepts and slopes to our models.

## 16. Null results due to hypothetical scenario?

- Could it be that you do not find a carryover effect of stress to preferences (in the form of risk attitudes), because you only prensent subjects to these (already) framed hypothetical contexts? I could imagine that people would have changed their risk-taking behavior when presented to more general gambles (i.e., without this DP trade offs, where a sure option vs. a risk option exists).

The problem of such a setting is that it does not measure risk preferences based on different levels as in other risk-eliciation settings (e.g., Holt and Laury, 2002; Eckel and Grossman, 2002, Dohmen et al., 2011).

Instead, people only face a binary choice, which dramatically limits the action space.

*** References

Bu, D., Hanspal, T., Liao, Y., & Liu, Y. (2020). Risk taking during a global crisis: Evidence from wuhan. Covid Economics, 5, 106-146.

Campos-Mercade, P., Meier, A. N., Schneider, F. H., & Wengström, E. (2021). Prosociality predicts health behaviors during the COVID-19 pandemic. Journal of Public Economics, 195, 104367.

Dohmen, T., Falk, A., Huffman, D., Sunde, U., Schupp, J., & Wagner, G. G. (2011). Individual risk attitudes: Measurement, determinants, and behavioral consequences. Journal of the european economic association, 9(3), 522-550.

Eckel, C. C., & Grossman, P. J. (2002). Sex differences and statistical stereotyping in attitudes toward financial risk. Evolution and human behavior, 23(4), 281-295.

Holt, C. A., & Laury, S. K. (2002). Risk aversion and incentive effects. American economic review, 92(5), 1644-1655.

Müller, S., & Rau, H. A. (2021). Economic preferences and compliance in the social stress test of the COVID-19 crisis. Journal of Public Economics, 194, 104322.

**Response:** Thank you for your suggestion. We agree that the measures of risk elicitation you cited can provide a more reliable and valid estimate of risk aversion and thus provide a better chance of finding an association with distress. However, we drew these hypotheses on studies that found such an association using exactly the Disease Problem (Druckman & McDermott, 2008; Lerner & Keltner, 2001). We thus cannot explain away our lack of positive results with the nature of the DP. We included this reasoning, and the references you suggested, in the discussion (ln. 591–597).

# References

American Psychological Association. (2014). *Stress in America Survey Methodology: 2013*. Https://Www.Apa.Org. https://www.apa.org/news/press/releases/stress/2013/methodology

American Psychological Association. (2019). *Stress in America^TM^ 2019: Interactive Graphics*. Https://Www.Apa.Org. https://www.apa.org/news/press/releases/stress/2019/interactive-graphics

Curran, P. G. (2016). Methods for the detection of carelessly invalid responses in survey data. *Journal of Experimental Social Psychology*, *66*, 4–19. https://doi.org/10.1016/j.jesp.2015.07.006

Druckman, J. N., & McDermott, R. (2008). Emotion and the Framing of Risky Choice. *Political Behavior*, *30*(3), 297–321. https://doi.org/10.1007/s11109-008-9056-y

Dupuis, M., Meier, E., & Cuneo, F. (2019). Detecting computer-generated random responding in questionnaire-based data: A comparison of seven indices. *Behavior Research Methods*, *51*(5), 2228–2237. https://doi.org/10.3758/s13428-018-1103-y

Galesic, M. (2006). Dropouts on the web: Effects of interest and burden experienced during an online survey. *Journal of Official Statistics*, *22*(2), 313–328.

Kahneman, D., & Tversky, A. (1984). Choices, values, and frames. *American Psychologist*, *39*(4), 341–350.

Klein, R. A., Ratliff, K. A., Vianello, M., Adams, R. B., Bahník, Š., Bernstein, M. J., Bocian, K., Brandt, M. J., Brooks, B., Brumbaugh, C. C., Cemalcilar, Z., Chandler, J., Cheong, W., Davis, W. E., Devos, T., Eisner, M., Frankowska, N., Furrow, D., Galliani, E. M., … Nosek, B. A. (2014). Investigating Variation in Replicability. *Social Psychology*, *45*(3), 142–152. https://doi.org/10.1027/1864-9335/a000178

Lerner, J. S., & Keltner, D. (2001). Fear, anger, and risk. *Journal of Personality and Social Psychology*, *81*(1), 146–159. https://doi.org/10.1037/0022-3514.81.1.146

Lieberoth, A., Lin, S.-Y., Stöckli, S., Han, H., Kowal, M., Gelpi, R., Chrona, S., Tran, T. P., Jeftić, A., Rasmussen, J., Cakal, H., Milfont, T. L., & The COVIDiSTRESS Global Survey Consortium. (2021). Stress and worry in the 2020 coronavirus pandemic: Relationships to trust and compliance with preventive measures across 48 countries in the COVIDiSTRESS global survey. *Royal Society Open Science*, *8*(2), 200589. https://doi.org/10.1098/rsos.200589

Sullivan, G. M., & Feinn, R. (2012). Using Effect Size—Or Why the P Value Is Not Enough. *Journal of Graduate Medical Education*, *4*(3), 279–282. https://doi.org/10.4300/JGME-D-12-00156.1

Yamada, Y., Ćepulić, D.-B., Coll-Martín, T., Debove, S., Gautreau, G., Han, H., Rasmussen, J., Tran, T. P., Travaglino, G. A., COVIDiSTRESS Global Survey Consortium, & Lieberoth, A. (2021). COVIDiSTRESS Global Survey dataset on psychological and behavioural consequences of the COVID-19 outbreak. *Scientific Data*, *8*(1), 3. https://doi.org/10.1038/s41597-020-00784-9
